# The Long-Term Functional and Oncologic Outcomes of Kidney-Sparing Surgery in Upper Tract Urothelial Carcinoma

**DOI:** 10.1245/s10434-024-16523-y

**Published:** 2024-11-16

**Authors:** Yu-Chieh Wang, Jian-Ri Li, Chuan-Shu Chen, Shian-Shiang Wang, Cheng-Kuang Yang, Kun-Yuan Chiu, Chiann-Yi Hsu

**Affiliations:** 1 Department of Medical Education, Taichung Veterans General Hospital, Taichung, Taiwan; 2 School of Medicine, Chung-Shan Medical University, Taichung, Taiwan; 3 Department of Urology, Department of Surgery, Taichung Veterans General Hospital, Taichung, Taiwan; 4 Department of Post-Baccalaureate Medicine, College of Medicine, National Chung Hsing University, Taichung, Taiwan, ROC; 5 College of Nursing, Hung-Kuang University, Taichung, Taiwan; 6 Institute of Medicine, Chung Shan Medical University, Taichung, Taiwan; 7 Department of Applied Chemistry, National Chi Nan University, Nantou, Taiwan; 8 Biostatistics Group, Department of Medical Research, Taichung Veterans General Hospital, Taichung, Taiwan

**Keywords:** Upper tract urothelial carcinoma, Transitional cell carcinoma, Nephroureterectomy, Organ-sparing treatment, Functional outcomes, Oncological outcomes

## Abstract

**Background:**

This study investigated the utilization of kidney-sparing surgery (KSS) as an alternative option to radical nephroureterectomy (RNU) in managing upper urinary tract urothelial carcinoma (UTUC) patients.

**Objective:**

Our study aimed to compare the functional outcomes and oncological outcomes between KSS and RNU.

**Material and Methods:**

We retrospectively analyzed 252 patients with UTUC without clinical node positivity or metastasis who had been treated with either RNU or KSS. We collected information on each patient, including clinicopathological factors, renal function variations, and oncological outcomes. Hemodialysis-free survival (HDFS), stage 4 chronic kidney disease (CKD4) progression-free survival (PFS), recurrence-free survival (RFS), and overall survival (OS) were assessed using inverse probability of treatment weighting (IPTW)-weighted Kaplan–Meier analysis. The hazard ratio for oncological and functional outcomes of KSS was analyzed using the Cox proportional hazards model.

**Results:**

The OS at 8 years was 77.06% (RNU) and 70.59% (KSS) and did not significantly differ between the two groups (*p* = 0.691), although the KSS group experienced a higher recurrence rate. Functional outcomes indicated no significant difference in postoperative renal function at 1 year; however, KSS was associated with better preservation of renal function (23.44% vs. 28.23%), albeit not statistically significant (*p* = 0.055). Kaplan–Meier analysis revealed no significant disparities in CKD4 PFS between the KSS and RNU groups involved in the study (*p* = 0.089), although the KSS group displayed poorer HDFS than the RNU group (*p* = 0.001).

**Conclusion:**

KSS had no compromising survival outcomes when compared with RNU, not only in low-risk patients but also in high-risk patients with a normal contralateral kidney. The efficacy of renal function preservation was presented in this study, however the results were below our expectations.

Upper urinary tract urothelial carcinomas (UTUCs) possess uncommon but lethal characteristics, with relatively modest survival outcomes among all urological malignancies.^1^ In comparison with bladder cancer, UTUCs are relatively rare, accounting for only 5–10% of UCs. However, their behavior appears aggressive, with 60% of patients presenting with invasive disease and 30% of patients presenting with metastatic disease at diagnosis.^2^

According to the current guidelines, kidney-sparing surgery (KSS) can be offered as an option in the management of low-risk UTUC diseases, which has reduced the morbidity rate associated with radical nephroureterectomy (RNU) without any inferior oncological outcomes.^3^ The recommended KSS options include tumor resection using an endoscopic approach; however, higher recurrence rates and the need for repeated interventions were notified.^4^ Segmental ureteral resection and distal ureterectomy with ureteroneocystostomy are both feasible treatments for ureteral urothelial carcinoma. The bench procedure with kidney autotransplantation can address tumors located in the pelvicalyceal system and is also regarded as an ultimate approach to avoid permanent loss of renal function.

Radical surgical treatments led to the deterioration of renal function at a rate as high as 25.1% postoperatively, as well as an increase in associated comorbidities.^5^ Impaired renal function eventually requires renal replacement therapy (RRT), leading to a poor quality of life (QoL), particularly in patients with solitary kidney disease or chronic kidney disease (CKD). Organ-sparing approaches provide particular benefits for patients diagnosed with tumor-bearing solitary kidneys or poor renal function.

However, KSS is only recommended in selected high-risk cases with imperative indications precluding RNU (e.g., renal insufficiency, solitary kidney, or bilateral disease).^6^ In the current healthcare environment, there has been an emphasis on shared decision making, with many patients expressing preferences regarding preserving the kidney or avoiding dialysis. KSS provides these patients with alternative treatment options. Evidence of high-risk characteristics managed by KSS is still lacking, and the selection criteria remain an issue that requires further investigation.

## Materials and Methods

### Study Population

In this study, we retrospectively reviewed the available clinical data obtained from Taichung Veterans General Hospital, a single tertiary referral center in central Taiwan. We reviewed patients diagnosed with UTUC with no evidence of distant metastasis followed by surgical treatment between January 2017 and December 2022. The exclusion criteria included patients who were clinical stage T3+, clinical lymph node-positive, undergoing neoadjuvant treatment, and/or receiving hemodialysis preoperatively. Finally, 252 patients were identified and were included in the analysis.

### Surgery

The decision regarding the use of either RNU or KSS was made based on the surgeon’s preference and the patient’s consent after discussions were held regarding the surgical approaches, prognosis, and kidney preservation. RNU, which included removal of the ipsilateral bladder cuff, was performed by experienced urological surgeons in our hospital. For KSS, surgical approaches depend on tumor location and size. For tumors located in the pelvicalyceal system, endoscopic ablation, partial nephrectomy, and a bench procedure with kidney autotransplantation were performed in our cases. Distal ureterectomy with ureteral re-implantation and segmental ureterectomy with primary end-to-end anastomosis are the preferred options for distal and middle-proximal ureteral tumors, respectively.

### Data Collection

The patients’ clinical characteristics were collected, including age, sex, body mass index (BMI), comorbidities, and renal function pre- and postoperatively. The standard follow-up protocol after surgery included analysis of laboratory data, urinalysis with urine cytology, chest plan film, and cystoscopy. Computed tomography was performed to identify local recurrence and distant organ metastasis. Patients were evaluated every 3 months for the first 2 years, every 6 months for the next 2 years, and annually thereafter. The primary endpoint of the study was the comparison of oncological outcomes (OS and recurrence-free survival [RFS]) and functional outcomes (hemodialysis-free survival [HDFS], stage 4 CKD [CKD4] progression-free survival [PFS]) between the RNU and KSS groups. OS was defined as the time from diagnosis to death or last follow-up due to any cause, and RFS was defined as the time from diagnosis to local recurrence in the tumor bed, intravesical recurrence, regional lymph node metastasis, distant lymph node metastasis, and organ metastasis.

### Statistical Analysis

Descriptive statistical analysis was conducted to compare the clinicopathological features, surgical details, and outcomes. Quantitative variables are expressed as mean with standard deviation (SD) or median with interquartile range (IQR), while categorical variables are reported as absolute values and percentages. Comparison of the data dispersion was performed using Student’s *t* test for continuous variables and either Fisher’s exact test or Pearson’s Chi-square test for categorical variables. The outcomes, including OS, RFS, CKD4 PFS, and HDFS, were all analyzed using the Kaplan–Meier method and were adjusted by inverse probability of treatment weighting (IPTW) to address the cofounding. Univariate and multivariate analyses with Cox proportional hazards models were used to assess the impact of various clinical and demographic factors on patient survival. For oncological outcomes, covariates were included in the model based on clinical relevance (high-risk features of UTUC) and statistical significance in univariate analysis. For functional outcomes, preoperative estimated glomerular filtration rate (eGFR) was a significant factor in both CKD4 progression and hemodialysis analysis, and was selected as one of the covariates. Estimated survival curves were plotted based on the fitted Cox model. In all statistical analyses, all tests were two-sided and statistical significance was set at *p* < 0.05.

## Results

### Clinical Demographic Characteristics

A total of 252 patients were included in the analysis, with 218 and 34 receiving RNU and KSS, respectively. Significant differences were found in patients who had solitary kidney disease (3.21% RNU vs. 14.71% KSS; *p* = 0.013), and a greater percentage of patients in the KSS group had bladder cancer (12.84% RNU vs. 35.29% KSS; *p* < 0.001). Differences in comorbidities were only found in cardiovascular disease (*p* = 0.007) and hypertension (*p* = 0.008). The preoperative eGFR of the KSS group was significantly poorer than that of the RNU group (46.06 vs. 54.58 mL/min/1.73 m^2^; *p* = 0.019). Overall, the study included 169 patients with advanced CKD stage (eGFR <60); 85.29% of patients in the KSS group and 64.22% in the RNU group (*p* = 0.015). All demographic characteristics are shown in Table 1.

**Table 1:** Clinical characteristics

	Total [*n* = 252]	Radical nephroureterectomy [*n* = 218]	Kidney-sparing surgery [*n* = 34]	*p*-Value
Age, years	69.63 (±10.27)	69.50 (±10.27)	70.44 (±10.35)	0.640
Sex [*n* (%)]				0.618
Male	116 (46.03)	99 (45.41)	17 (50.00)	
Female	136 (53.97)	119 (54.59)	17 (50.00)	
BMI, kg/m^2^	24.31 (±4.41)	24.31 (±4.46)	24.43 (± 4.10)	0.914
Comorbidity [*n* (%)]				
Diabetes mellitus	86 (34.13)	74 (33.94)	12 (35.29)	0.877
Hyperlipidemia	56 (22.22)	51 (23.39)	5 (14.71)	0.257
Hypertension	96 (38.10)	90 (41.28)	6 (17.65)	0.008
CVD	150 (59.52)	137 (62.84)	13 (38.24)	0.007
COPD	24 (9.52)	21 (9.63)	3 (8.82)	1.000
Liver disease	10 (3.97)	8 (3.67)	2 (5.88)	0.629
PVD	12 (4.76)	10 (4.59)	2 (5.88)	0.668
Gout	32 (12.70)	28 (12.84)	4 (11.76)	1.000
BPH	65 (25.79)	56 (25.69)	9 (26.47)	1.000
CTD	12 (4.76)	12 (5.50)	0 (0)	0.378
Solitary kidney disease [*n* (%)]	12 (4.76)	7 (3.21)	5 (14.71)	0.013
Concomitant bladder cancer [*n* (%)]	40 (15.87)	28 (12.84)	12 (35.29)	0.001
Preoperative creatinine, mg/dL	1.52 (±1.07)	1.51 (±1.13)	1.59 (±0.63)	0.013
Preoperative eGFR, mL/min/1.73 m^2^	53.43 (±22.37)	54.58 (±22.86)	46.06 (±17.50)	0.019
Preoperative CKD stage [*n* (%)]				0.015
I–II	83 (32.94)	78 (35.78)	5 (14.71)	
III–V	169 (67.06)	140 (64.22)	29 (85.29)	

*BMI* body mass index, *CVD* cardiovascular disease, *COPD* chronic obstructive pulmonary disease, *PVD* peripheral vascular disease, *BPH* benign prostate hyperplasia, *CTD* connective tissue disease, *eGFR* estimated glomerular filtration rate, *CKD* chronic kidney disease

### Tumor Characteristics

The KSS group was predominantly ureteral cancer (76.47%), while on the other hand, the majority of RNU cases were made up of ureter and renal pelvis tumors of approximately equal proportions. Four of the 34 KSS cases were bilateral tumors, a significantly greater percentage than that of the RNU group (*p* = 0.001). Significant differences were also found in tumor size (RNU 2.39 vs. KSS1.71 cm; *p* = 0.013) and percentage of hydronephrosis (39.45% RNU vs. 64.71% KSS; *p* = 0.006). The distribution of clinical stages did not differ between the two groups. Overall, 78/252 (30.95%) patients were diagnosed with pathological stages >T3, with no significant differences between the two groups. High-grade disease was identified in 85.32% of all tumors, with significant differences being noted in the distribution of tumor grade (*p* = 0.017). The tumor characteristics are shown in Table 2.

**Table 2:** Tumor characteristics

	Total [*n* = 252]	Radical nephroureterectomy [*n* = 218]	Kidney-sparing surgery [*n* = 34]	*p*-Value
Primary tumor site^a^ [*n* (%)]				<0.001
Ureter	114 (45.24)	87 (39.91)	26 (76.47)	
Renal pelvis	101 (40.08)	98 (44.95)	4 (11.76)	
Calyx	17 (6.75)	15 (6.68)	2 (5.58)	
Multiple	20 (7.94)	18 (8.26)	2 (5.58)	
Size on image, cm	2.29 (±1.44)	2.39 (±1.47)	1.71 (±1.06)	0.013
Multifocality [*n* (%)]	38 (15.08)	32 (14.68)	6 (17.65)	0.653
Clinical T stage [*n* (%)]				0.264
cTa	119 (47.22)	99 (45.41)	20 (58.82)	
cT1	53 (21.03)	46 (21.10)	7 (20.59)	
cT2	80 (31.75)	73 (33.49)	7 (20.59)	
Hydronephrosis [*n* (%)]	108 (42.86)	86 (39.45)	12 (64.71)	0.006
*Pathology characteristics*				
Pathology T stage [*n* (%)]				0.045
pTis	11 (4.37)	9 (4.13)	2 (5.88)	
pTa	22 (8.72)	15 (6.88)	7 (20.59)	
pT1	102 (40.48)	94 (43.12)	8 (23.53)	
pT2	39 (15.48)	33 (15.14)	6 (17.65)	
pT3	78 (30.95)	67 (30.73)	11 (32.25)	
Pathology N stage [*n* (%)]				0.008
pN+	4 (1.59)	1 (0.46)	3 (8.82)	
pN-	248 (98.41)	217 (99.54)	31 (91.18)	
Tumor grade [*n* (%)]				0.017
Low	37 (14.68)	27 (12.39)	10 (29.41)	
High	215 (85.32)	191 (87.61)	24 (70.59)	
LVI+ [*n* (%)]	38 (15.08)	34 (15.60)	4 (11.76)	0.561
Margin+ [*n* (%)]	8 (3.17)	5 (2.29)	3 (8.82)	0.078
Micropapillary UC+ [*n* (%)]	2 (0.79)	1 (0.46)	1 (2.94)	0.252
Concomitant CIS+ [*n* (%)]	35 (13.89)	32 (14.68)	3 (8.82)	0.437

*LVI* lymphovascular invasion, *UC* urothelial carcinoma, *CIS* carcinoma in situ

^a^Location is based on the highest tumor volume

### Survival Outcomes

**Fig. 1:**
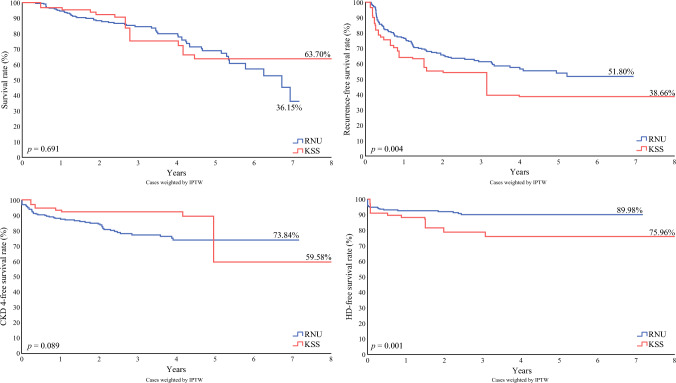
Oncologic and functional survival for RNU and KSS with the IPTW-weighted Kaplan–Meier method. Numbers at risk after 8 years are as follows: overall survival—number at risk RNU 36.15 and number at risk KSS 63.70, log-rank *p* = 0.691; recurrence—number at risk RNU 51.80 and number at risk KSS 38.66, log-rank *p* = 0.004; CKD4 progression-free survival—number at risk RNU 73.84 and number at risk KSS 59.58, log-rank *p* = 0.089; HD-free survival—number at risk RNU 89.98 and number at risk KSS 75.96, log-rank *p* = 0.001. *KSS* kidney-sparing surgery, *RNU* radical nephroureterectomy, *CKD4* stage 4 chronic kidney disease, *HD* hemodialysis, *IPTW* inverse probability of treatment weighting

**Fig. 2:**
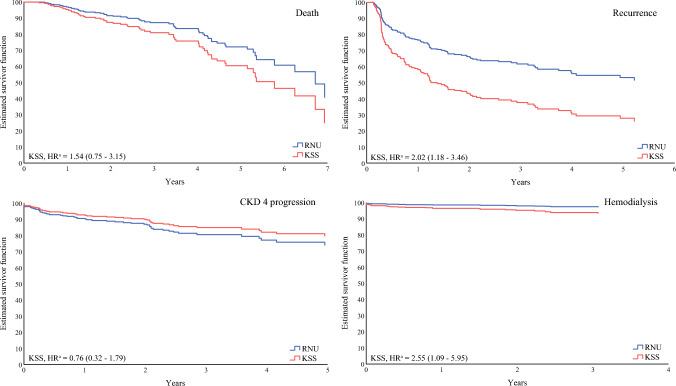
Estimated survival curves using the Cox proportional hazard model. HR of death 1.54, 95% CI 0.75–3.15, *p* = 0.235; HR of recurrence 2.02, 95% CI 1.18–3.46, *p* = 0.01; HR of CKD4 progression 0.76, 95% CI 0.32–1.79, *p* = 0.527; HR of hemodialysis 2.55, 95% CI 1.09–5.95, *p* = 0.001. *KSS* kidney-sparing surgery, *RNU* Radical nephroureterectomy, *HR* hazard ratio, *CKD4* stage 4 chronic kidney disease, *CI* Confidence interval

In the overall analysis, the median overall survival (OS) time was 2.84 years (IQR 1.91–4.58), with no significant difference between the KSS and RNU groups (RNU 2.85 years vs. KSS 2.73 years; *p* = 0.549). OS at 8 years was 77.06% (RNU) and 70.59% (KSS), with no significant difference (*p* = 0.465). IPTW-weighted Kaplan–Meier estimation with a log-rank test revealed no significant disparities in OS between the two groups in our study (*p* = 0.691) (Fig. 1). After adjusting for confounding factors, multivariable Cox regression analysis indicated that KSS was associated with an HR of 1.54 (95% CI 0.75–3.15; *p* = 0.235) in OS, as shown in Table 3. The median time to recurrence did not show a significant difference between the two groups (KSS 2.15 years vs. RNU 1.37 years; *p* = 0.059). Tumor recurrence was observed in 39.93% of the RNU group and 55.88% of the KSS group (*p* = 0.079). However, analysis of RFS using IPTW-weighted Kaplan–Meier estimation revealed a significant difference between the two groups (log rank *p* = 0.004) (Fig. 1). Similarly, multivariable Cox regression analysis revealed a hazard ratio (HR) of 2.02 (95% CI 1.18–3.46; *p* = 0.010), as shown in Table 3. The estimated survival curves of OS and RFS using the Cox proportional hazard model suggested better survival in the RNU group, however the HR indicated no significant difference in OS (Fig. 2)

**Table 3:** Multivariable Cox regression analysis of overall survival and recurrence-free survival

Variables	Overall survival			Recurrence-free survival		
	HR	95% CI	*p-*value	HR	95% CI	*p-*Value
KSS	1.54	0.75–3.15	0.235	2.02	1.18–3.46	0.01
Age	1.05	1.02–1.08	<0.001	1.00	0.98–1.02	0.884
Laterality (bilateral vs. unilateral)	3.32	1.01–10.92	0.048	0.54	0.15–1.90	0.337
LVI	2.20	1.21–3.99	0.010	1.03	0.6–1.77	0.920
Multifocality	1.64	0.82–3.26	0.159	1.89	1.14–3.13	0.014
Tumor grade (high vs. low)	2.43	0.93–6.36	0.071	1.25	0.71–2.19	0.437

*HR* hazard ratio, *CI* confidence interval, *KSS* kidney-sparing surgery, *LVI* lymphovascular invasion

### Functional Outcomes

The mean postoperative eGFR of the KSS and RNU groups showed no significant difference at 1 year (KSS 40.86 vs. RNU 38.83 mL/min/1.73 m^2^; *p* = 0.466) (Table 4). The overall median decline in the eGFR rate 1-year postoperatively was 23.80%. The KSS group showed better preservation of renal function, with only 16.45% decline in eGFR compared with 24.96% in the RNU group, but with no significant difference (*p* = 0.055). The median time to CKD4 was not significantly different (RNU 30.49 vs. KSS 25.54 months; *p* = 0.620). No significant disparities in CKD4 PFS were observed between the KSS and RNU groups (*p* = 0.089) (Fig. 1). Multivariable Cox regression analysis revealed an HR of 0.76 (95% CI 0.32–1.79; *p* = 0.527) (Table 5). Overall, 26 (10.32%) patients within the study eventually underwent hemodialysis. Significant differences were found in the percentage of patients who underwent hemodialysis (*p* = 0.013). The median time to HD was not significantly different between the two groups (RNU 33.13 vs. KSS 27.12 months; *p* = 0.351), however, analysis of HDFS using the IPTW-weighted Kaplan–Meier method showed superior results for the RNU group (log rank *p* = 0.006) (Fig. 2). Multivariate analysis also found that KSS was an independent risk factor of hemodialysis (HR 2.55, 95% CI 1.09–5.95; *p* = 0.001) (Table 5). The estimated survival curves of CKD4 PFS showed better functional preservation results in the KSS group, however worse HDFS was observed in the study (Fig. 2)

**Table 4:** Renal function preservation

	Total [*n* = 252]	Radical nephroureterectomy [*n* = 218]	Kidney-sparing surgery [*n* = 34]	*p-*Value
Postoperative creatinine, mg/dL	2.02 (±1.63)	1.96 (±1.53)	2.46 (±2.26)	0.312
Postoperative eGFR, mL/min/1.73 m^2^	40.63 (±17.57)	40.86 (±17.07)	38.83 (±21.29)	0.466
Change of creatinine, %	38.01 (±49.42)	37.85 (±39.28)	37.85 (±91.51)	0.054
Change of eGFR, %	−23.80 (±23.87)	−24.96 (±22.76)	−16.45 (±29.28)	0.055
Time to CKD4, months	29.68 (IQR 16.36–50.69)	30.49 (IQR 16.43–50.85)	25.54 (IQR 12.39–50.41)	0.620
Time to HD, months	32.72 (IQR 19.33–54.14)	33.13 (IQR 20.14–54.6)	27.12 (IQR 16.53–52.16)	0.351

*eGFR* estimated glomerular filtration rate, *CKD4* stage 4 chronic kidney disease, *HD* hemodialysis, *IQR* interquartile range

**Table 5:** Multivariable Cox regression analysis of CKD4 progression-free survival and hemodialysis-free survival

Variables	CKD4 progression- free survival			Hemodialysis-free survival		
	HR	95% CI	*p*-Value	HR	95% CI	*p-*Value
KSS	0.76	0.32–1.79	0.527	2.55	1.09–5.95	0.031
Age	1.04	1.01–1.07	0.021	0.98	0.94–1.03	0.466
Preoperative eGFR, mL/min/1.73 m^2^	0.98	0.97–0.99	0.003	0.91	0.88–0.94	<0.001

*CKD4* stage 4 chronic kidney disease, *HR* hazard ratio, *CI* confidence interval, *KSS* kidney-sparing surgery, *eGFR* estimated glomerular filtration rate

## Discussion

As functional preservation and QoL have become increasingly valued among cancer patients, KSS is being scrutinized more closely. To the best of our knowledge, our study is the only series to compare survival outcomes between KSS and RNU based on highly selective criteria (cT2−, cN0). Additionally, this is the first study to compare renal preservation efficacy based on CKD4 PFS and HDFS, which has been presented as an innovative method.

Several studies have validated the efficacy of KSS in UTUC and provided positive findings on oncologic outcomes. Seisen et al. performed a systematic review involving KSS versus RNU, where no significant difference was found in terms of cancer-specific survival (CSS) and other oncologic outcomes. Endoscopic treatments experienced similar CSS when compared with RNU, but an increased risk of local recurrence has been reported.^3^ Selection bias for favorable tumor characteristics (low-stage disease, etc.) in the KSS group remained a major concern in this study. Hendriks et al. analyzed survival and matched propensity weights based on risk stratification and showed comparable results for all survival outcomes;^7^ however, in their study, 86.5% of the KSS group had stage Ta disease, which contributed to a significant difference compared with the RNU group. In our study, we selected patients based on their clinical stage, rather than pathologic stage, to simulate real-life clinical scenarios, with no significant difference in staging distribution. With an even distribution of high-risk patients in the two groups, we eliminated the selection bias of risk distribution. Finally, our OS showed a non-inferior outcome of KSS, with results comparable with those of previous studies, despite a higher percentage of high-risk patients in both groups.^8–10^ For the issue of potential confounding variables or covariates that may influence survival outcomes, Cox regression analysis was also conducted and showed similar results. The current consensus of candidates for KSS was found to be widespread, based on the results of our study and the studies mentioned above.

Tumor recurrence remains an important issue after surgery in patients with UTUC. Despite complete removal of the entire ipsilateral ureter with a functional kidney, the intravesical recurrence rate has been reported to be 22–47% even when treated with a radical approach.^11^ Higher recurrence rates, reportedly as high as 90% in the endoscopic approach, vary widely. Similarly, wide-ranging recurrence rates were reported in segmental ureterectomy, i.e. from 28% to 69%.^12–14^ Luo et al. performed a systematic review involving five survival outcomes, with results showing significantly poorer RFS with KSS (HR 1.61, 95% CI 1.03–2.51; *p* = 0.04).^15^ Comparable results were observed in our cohort and intravesical recurrence accounted for the majority of recurrence events. The higher recurrence rate observed in the KSS group can be reasonably explained by the risks associated with the KSS strategy, particularly in patients treated with the ureteroscopic approach. Sharma et al. also analyzed a retrospective study and found that patients undergoing URS had an increased intravesical recurrence rate (HR 1.40; *p* = 0.04).^16^

However, intravesical recurrence can be properly managed using subsequent transurethral resection of bladder tumors and long-term surveillance to mitigate morbidity. Prevention of bladder tumor recurrence through the use of one intravesical instillation after RNU has been recommended by several studies, including two prospective studies.^17,18^ While there is currently no evidence to support the use of intravesical instillation after KSS, this strategy should be effective in a similar setting and is worthy of further study. Surgical treatment, along with long-term strict surveillance, is mandatory in patients with KSS, particularly those diagnosed with high-risk tumors.

The preservation of kidney function and avoidance of renal replacement treatment have received widespread attention with regard to the management of UTUC, and we consider this as one of the primary objectives of this study. The major disadvantage of RNU is the development of CKD or the progression of renal insufficiency postoperatively. The decline in renal function following surgery has not only contributed to long-term complications and increased mortality but has also significantly affected the patients’ QoL.^19^ Another important issue is the limited efficacy of adjuvant treatment after RNU. Kaag et al. conducted a retrospective study of 388 UTUC patients and reported a mean decline in eGFR of 24%; 35% of patients lost their eligibility for cisplatin-based chemotherapy after RNU.^20^ Lane et al. also reported a 24% decline in patients who were eligible for cisplatin-based chemotherapy post-RNU (*p* < 0.001).^21^ In our study, the benefits of renal function preservation did not meet our anticipated results. Two possible reasons for this include significantly poorer preoperative renal function in the KSS group, as well as the higher proportion of patients with advanced CKD in the KSS group, which may have obscured the preservation effect. Preoperative eGFR was selected as one of the covariables of functional outcome in the multivariate Cox proportional hazard model, based on the results of the univariate analysis. KSS demonstrated an HR of 0.76 in CKD4 PFS, and the estimated survival curves of CKD4 PFS showed better functional preservation results in the KSS group. Another reason may be the higher proportion of solitary kidney disease observed in the KSS group, possibly contributing to the below-expectation results. Kim et al. analyzed the association between solitary kidney disease and CKD and reported an HR of 3.26 (95% CI 1.63–6.54) for CKD in the solitary kidney disease group.^22^ One potential solution is to match patients based on their renal function or CKD stages. By comparing groups with similar preoperative renal function conditions, we may better elucidate the actual benefits of renal function preservation. There are still certain noteworthy highlights resulting from our study: two of four patients with bilateral disease and all five solitary kidney disease patients in the KSS group avoided hemodialysis until the final follow-up or before death. By choosing KSS as a treatment modality, renal function will potentially be spared, and this might prevent dialysis in the case of a solitary kidney. These results represent both the success and objective of organ preservation in selected patients.

In our study, we excluded UTUC patients at clinically locally advanced (pT3+ and pN+) stages who were expected to have a poor prognosis.^23^ The cut-off point for clinical stage was based on its significance in predicting poor survival outcomes in patients with UTUC at stages T3+ or those with positive lymph node involvement, while considering adjuvant chemotherapy would be necessary for improving survival. Multivariate analysis taken from our study showed that clinical T stage had no significant effect on OS, however those patients who had received either adjuvant chemotherapy or immunotherapy experienced poorer outcomes. This could be attributed to the fact that patients requiring adjuvant chemotherapy tend to have reached a higher stage or possess unfavorable pathologic tumor characteristics (positive margin, lymphovascular invasion, etc.). However, whether the adverse effects of adjuvant treatment lead to a poor prognosis should be further investigated. Our Cox regression analysis also showed that hyperthyroidism was a significant risk factor for poor survival prognosis (HR 8.08, 95% CI 1.90–34.40; *p* = 0.005), and hyperlipidemia was associated with better OS outcomes (HR 0.24, 95% CI 0.1–0.6; *p* = 0.002). The association between thyroid disease and UTUC still lacks sufficient study. Riis et al. analyzed a register-based study that revealed that patients with hyperthyroidism experienced an increased risk of cancer, particularly lung, prostate, and breast cancer, although outcome analysis of their cancer patients was not conducted.^24^ Dyslipidemia can significantly affect and contribute to poor outcomes in cancer patients in various ways, including promoting tumor invasion and metastasis, producing resistance to cancer drugs, and increasing the cardiac-vascular toxicity of anticancer regimens.^25^ Conversely, dyslipidemia was associated with better survival outcomes in our study. The use of statin medication in dyslipidemia patients may have been related to these results. Yang et al. conducted a meta-analysis and reported that the use of statins was associated with a decline in both CSS (HR 0.78, 95% CI 0.74–0.84) and RFS (HR 0.87, 95% CI 0.78–0.97);^26^ however, the specific cancer type was not demonstrated in the study and the association of urothelial carcinoma was not mentioned. Lim et al. investigated the impact of statin use on prognosis following RNU for UTUC but reported no improvement in RFS, CSS, or OS associated with statin use.^27^ Future studies could focus on whether statins or other lipid-lowering medications can improve the survival outcomes of UTUC patients.

This study has several limitations. First, its retrospective nature and non-randomized analysis may have contributed to lower levels of evidence. Second, the risk of selection bias in treatment modality selection was inevitable in our study, despite our use of matching analysis with IPTW and analysis of the HR with the Cox proportional hazards model. Patients with solitary kidney disease or a strong preference to avoid hemodialysis tend to choose KSS as their treatment option. Additionally, unmeasured confounders that may influence both the treatment choice and the outcome can leave the analysis vulnerable to bias. Furthermore, the current diagnostic tools, although highly sensitive, are not entirely accurate in determining tumor staging. Finally, we acknowledge the relatively small number of patients in the KSS group compared with the RNU group, which may affect the significance of our results.

## Conclusion

Based on our results, KSS is a viable option for patients with UTUC, not only in low-risk patients but also in high-risk patients with a normal contralateral kidney. This study has underscored the potential benefits of KSS in preserving renal function. Although the KSS group had a higher recurrence rate, proper management involving strict surveillance and potential intravesical instillation can overcome these shortcomings. KSS demonstrates itself as a treatment modality offering an increased emphasis on both functional preservation and QoL in the field of cancer management.

## Data Availability

The datasets generated and/or analyzed during the current study are available from the corresponding author on reasonable request.
